# Supplementary material for: Charge transfer drives anomalous phase transition in ceria
Source: Nat Commun. 2018 Nov 29;9:5063. doi: 10.1038/s41467-018-07526-x (PMC6265291; doi:10.1038/s41467-018-07526-x)
Supplement: Supplementary file 1 — Supplementary Information [file 41467_2018_7526_MOESM1_ESM.pdf]

Supplementary Information

Charge transfer drives anomalous phase transition in ceria

Zhu et al.

Supplementary Figures

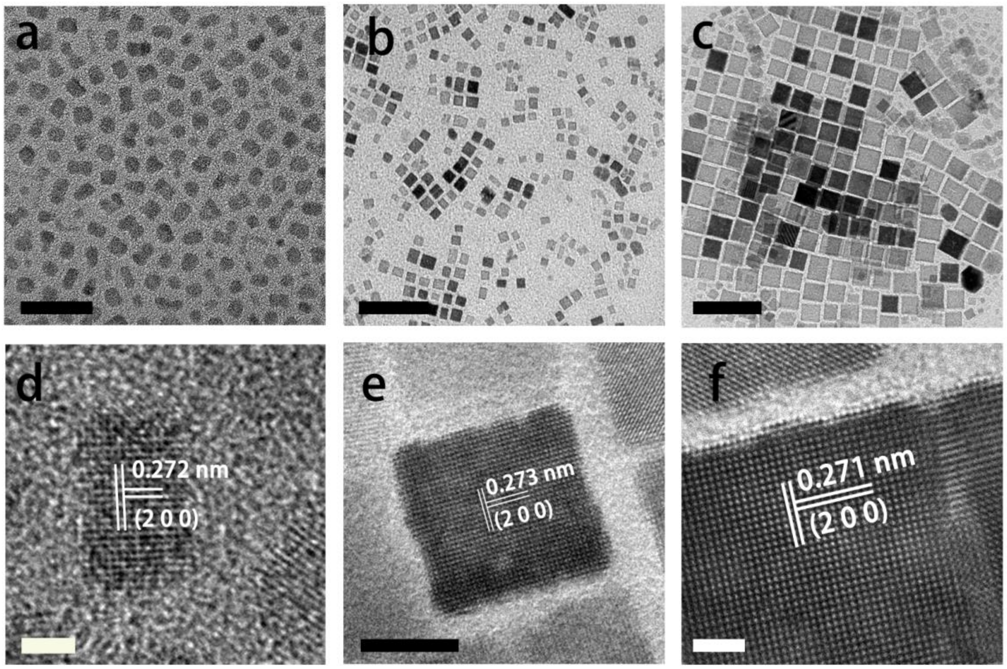

Supplementary Figure 1 | Morphology of the ceria nanoparticles. (a-c) TEM images of monodispersive CeO_2 nanocubes with particle sizes of 5 nm, 9 nm and 18 nm, respectively. The scale bars are 20 nm, 50 nm and 50 nm, respectively. The exposed (2 0 0) surfaces could be stabilized by oleic acid. **(d-f)** High resolution TEM images of the related CeO_2 nanoparticles. The scale bars are 2 nm, 5 nm and 2 nm, respectively. The interplanar crystal spacings corresponding to (2 0 0) are shown on the images.

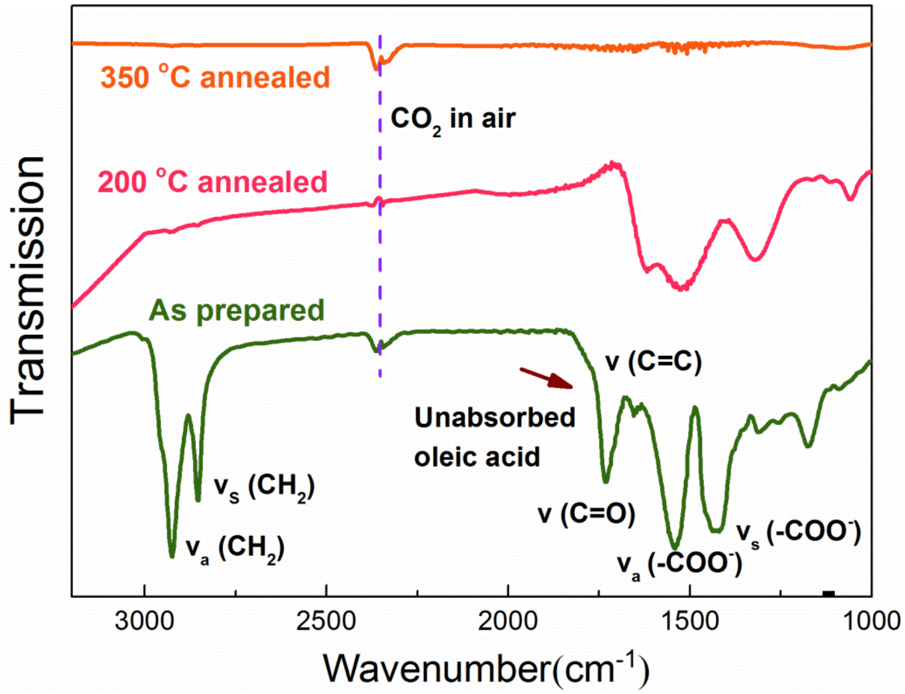

Supplementary Figure 2│FT-IR spectra of 5 nm ceria after annealed at different temperatures (see Supplementary Note 1 for details)**.** The vertical purple line shows the peak position of CO_2 in air, and the brown arrow shows the peaks representing the unabsorbed oleic acid.

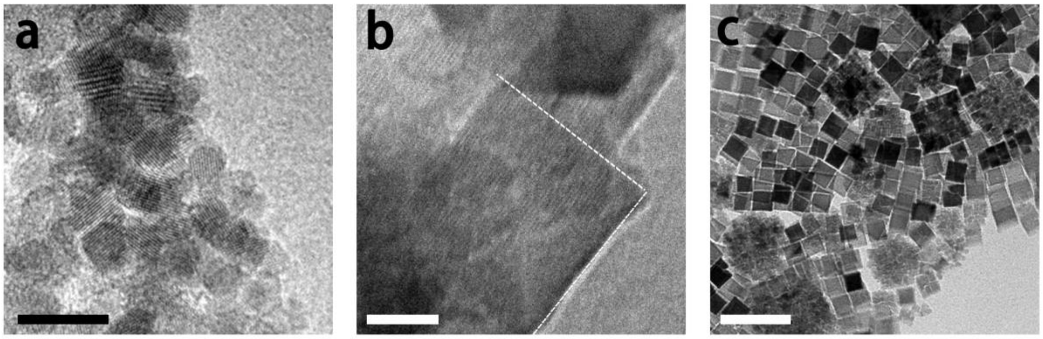

Supplementary Figure 3 | Morphology of nanosized ceria after annealing in air at 350 ^{o}C. (a - c) TEM images of annealed CeO_2 with the sizes of 5 nm, 9 nm and 18 nm, respectively. The white dash line emphasizes the edges of the nanocubes. The scale bars are 10 nm, 5 nm and 100 nm, respectively.

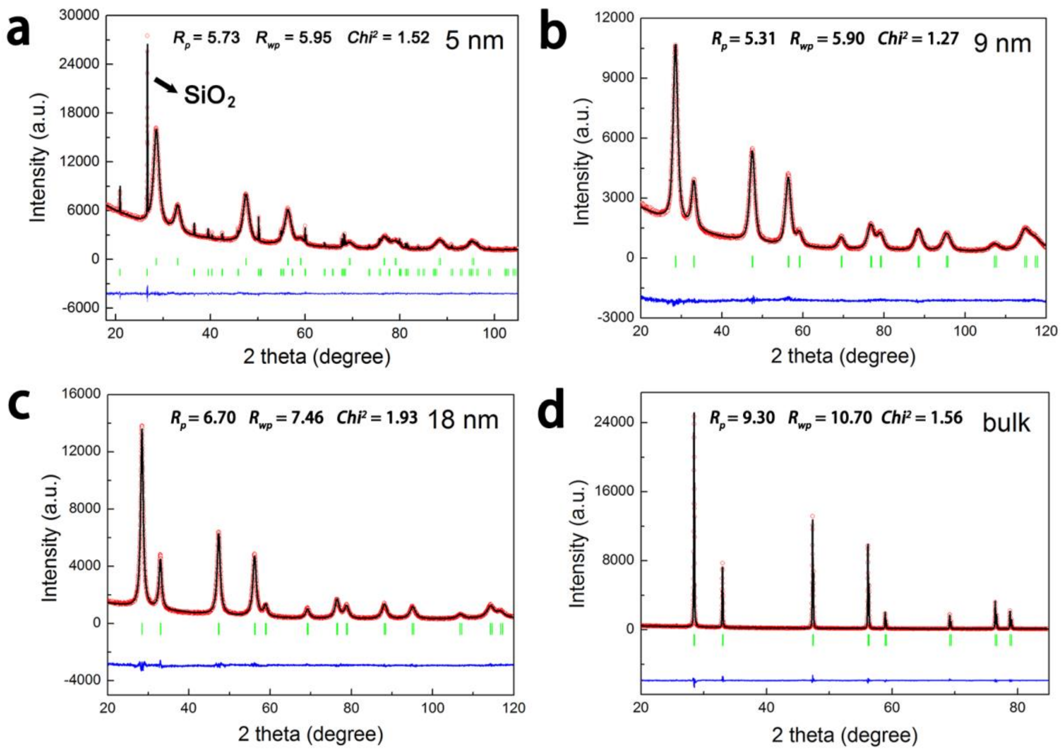

Supplementary Figure 4│Examples of Rietveld refinement for the samples of (a) 5 nm, **(b)** 9 nm, **(c)** 18 nm and **(d)** bulk taken at room temperature. The quartz internal standard has been mixed with the 5 nm ceria for calibration. The raw experimental data are shown with the red hollow circles. The black lines are the calculated results based on the *Fm*-3*m* space group. The green vertical lines show the peak positions. The blue lines show the difference between the raw data and the calculated patterns.

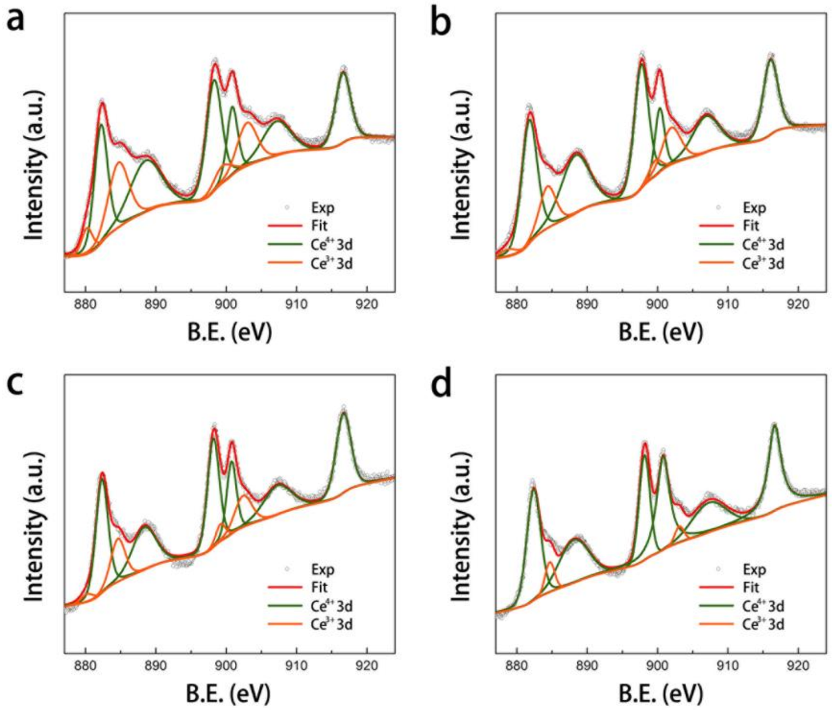

Supplementary Figure 5│XPS spectra for ceria in different sizes of (a) 5 nm, **(b)** 9 nm, **(c)** 18 nm and **(d)** bulk. Detailed analysis is presented in Supplementary Note 2.

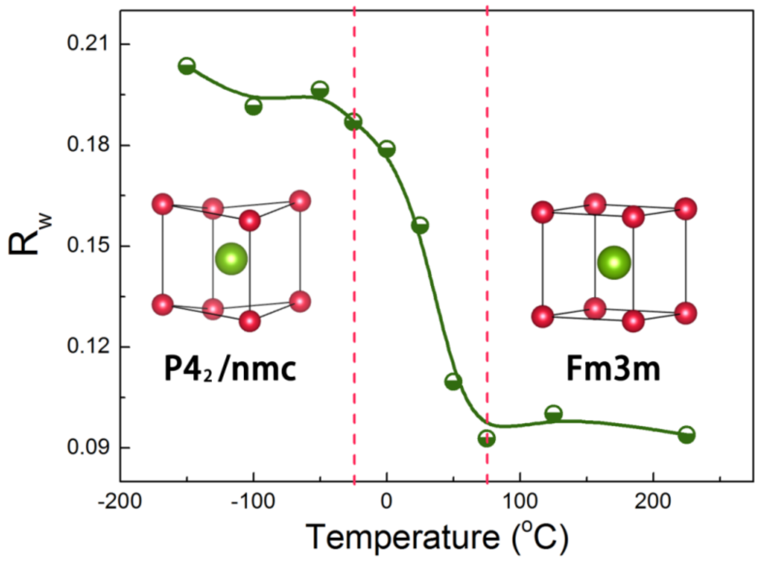

Supplementary Figure 6 │ Temperature dependence of the agree factor (R_w) of the low-*r* nPDF refinement using cubic-fluorite model. The green circles are the R_w values obtaining from the low-*r* nPDF refinements, and the green line shows the trend of the R_w decrease. The vertical red dash line shows the temperature range of transition from -25 °C to 75°C. The insets are the schematic diagrams depicting the correspond structure in the specific temperature ranges.

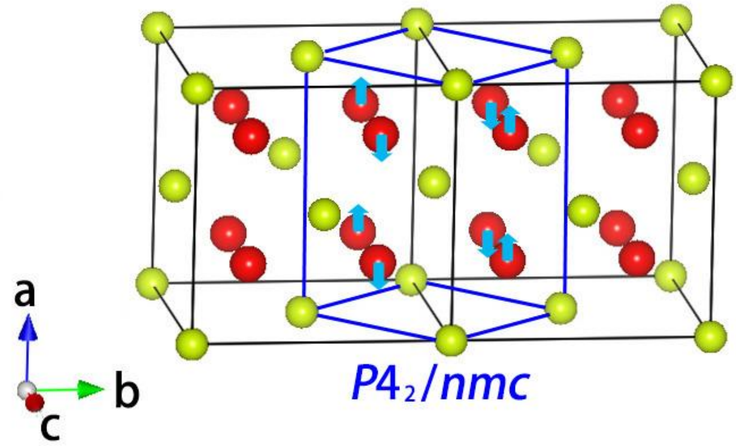

Supplementary Figure 7 | The conversion relation between cubic and tetragonal unit cell. The cubic unit cells are depicted in black lines and the tetragonal unit cell is depicted in blue lines. The arrows represent the oxygen displacements in tetragonal phase.

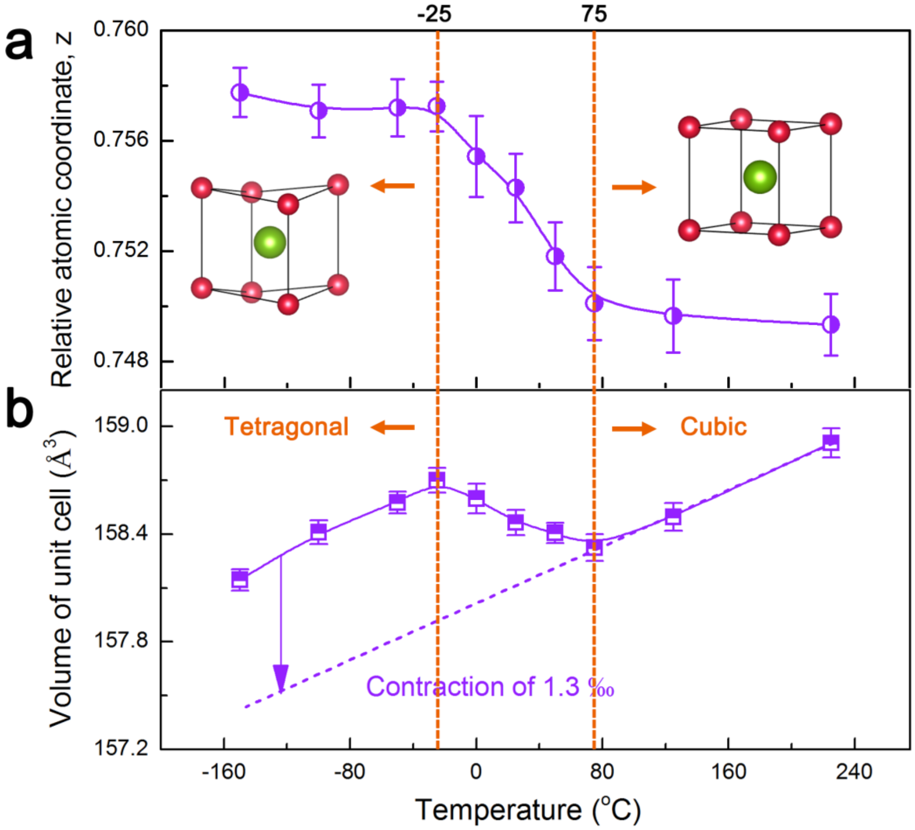

Supplementary Figure 8│Detailed results of the nPDF refinements. (a) Relative atomic coordinates of oxygen sites as a function of temperature. **(b)** Volume of the unit cell as a function of temperature. All the error bars are obtained from the fitting results with PDFgui software. In this figure, an expanded unit cell, whose volume is two times larger than the original $P4_2/nmc$ structure, has been utilized for the tetragonal phase at low temperature. This makes the volumes of the tetragonal and cubic phase comparable. We can see that a volume contraction is observed during the tetragonal to cubic phase transition, which is consistent with the XRD results.

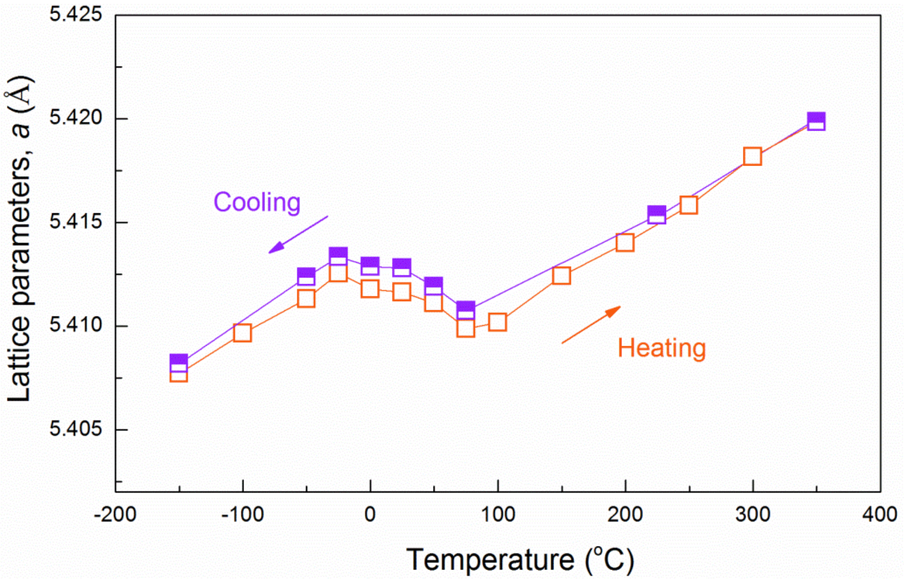

Supplementary Figure 9 | Lattice parameters of *a*-axis extracted from XRD results under temperature cycling condition. Since XRD method cannot distinguish the tetragonal and cubic ceria due to the peak broadening and overlapping, we adopted cubic *Fm*-3*m* model for the refinements. The subtle difference between the lattice constants upon heating and cooling indicates that the observed phase transition in nanosized ceria is reversible.

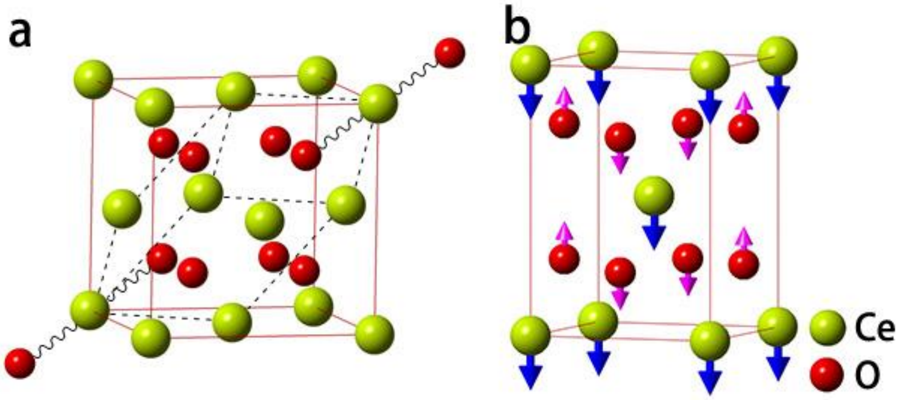

Supplementary Figure 10│Schematic diagram of the Raman vibration mode. (a) Schematic diagram of triply degenerate vibration mode (F_{2g}) along (1 1 1) direction in fluorite structure. The dash line depicts the primitive cell of CeO_2. **(b)** Schematic diagram of the vibration mode correlated with the opposite vibration of Ce and O atoms along *c*-axis.

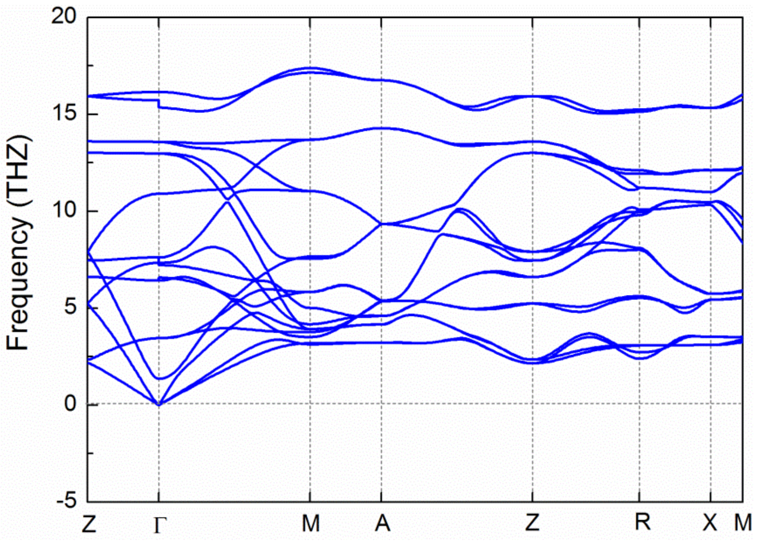

Supplementary Figure 11 | The stable phonon dispersion of the *P*4$_2$/*nmc* structure obtained by modulating the positions of the oxygen anions along the direction of the *M*-point soft phonons. The high-symmetry points of the Brillouin zone are denoted as Γ (0 0 0), M (0.5 0.5 -0.5), X (0 0 0.5), P (0.25 0.25 0.25), N (0 0.5 0).

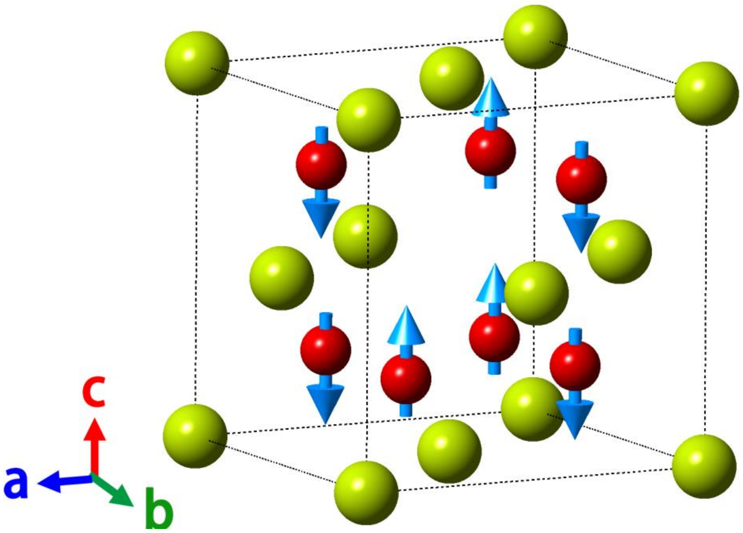

Supplementary Figure 12| The schematic diagram of the oxygen displacement for the relaxed tetragonal structure containing one oxygen vacancy. The blue arrows show the directions of oxygen displacements.

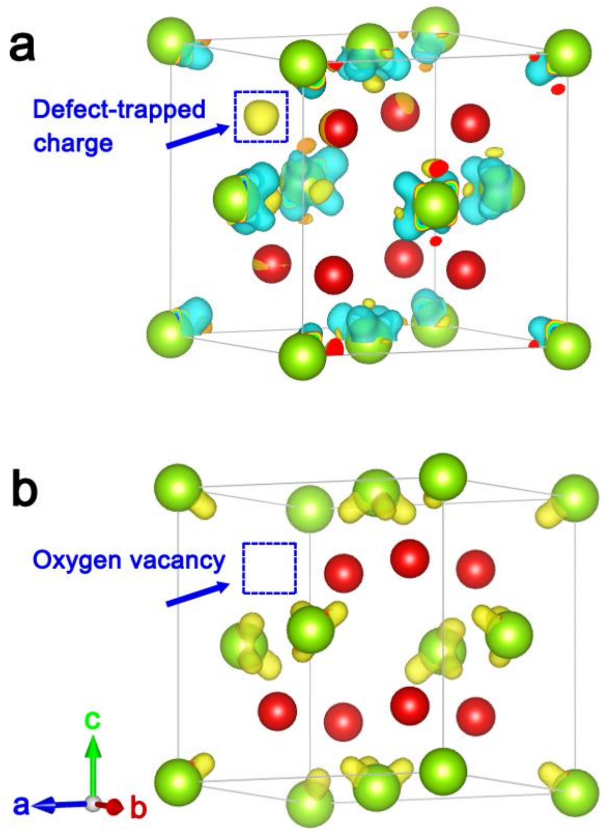

Supplementary Figure 13 | The spin charge density of **(a)** tetragonal phase and **(b)** cubic phase with one oxygen vacancy in their unit cells. Notably, excess spins were found in the vacancy of tetragonal lattice, while no excess spins were found in the vacancy of cubic lattice. The calculated results accord well with the EPR experiment (Fig. 4a-b), and we suppose the distinction of charge states could play a key role in the stability of different configurations.

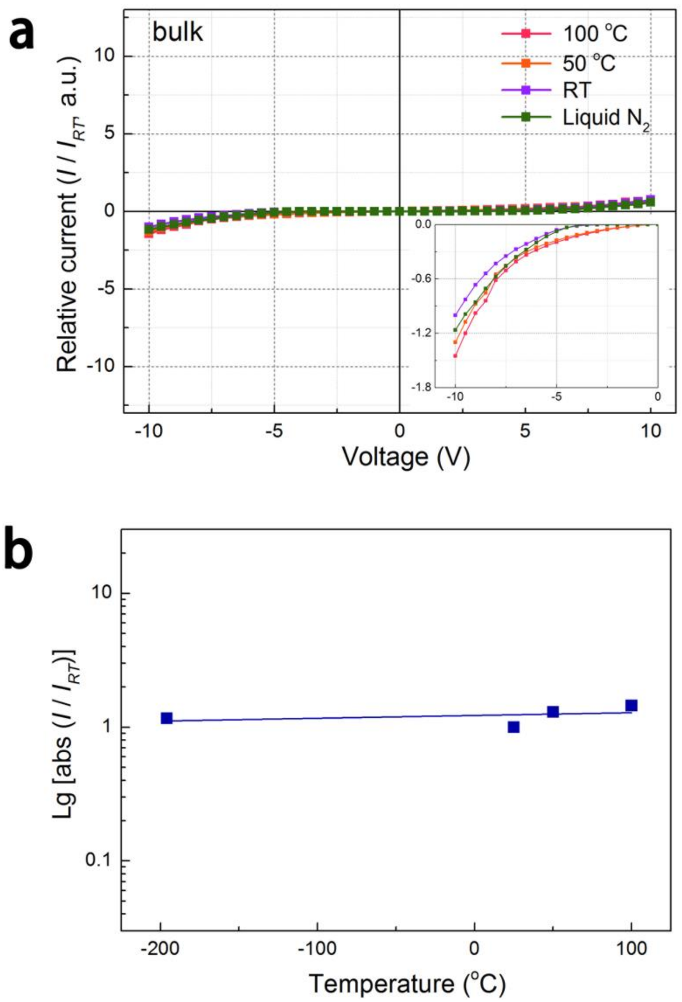

Supplementary Figure 14│The current-voltage characteristic for the bulk ceria. (a) The *I-V* curves for the bulk sample under varying temperature conditions. The inset depicts the expanded low current region. **(b)** Temperature dependence of the log-scaled relative currents with the applied voltage of 10 V.

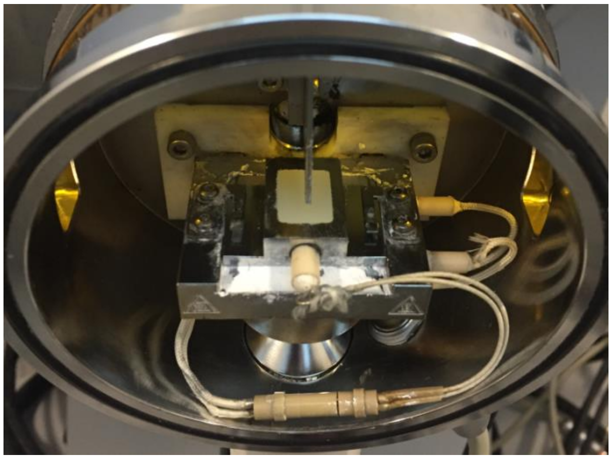

Supplementary Figure 15 | The configuration of the low temperature attachment of PW 3040-X'Pert Pro diffractometer.

Supplementary Notes

Supplementary Note 1

The result of the FT-IR analysis indicates that the as-prepared 5 nm ceria are coated with oleic acid. The bands around 2926 cm^{-1} and 2850 cm^{-1} are for the symmetric and asymmetric CH_2 stretching mode, respectively[1]. The small peak at around 1649 cm^{-1} is due to the C=C stretching mode. And the peaks around 2352 cm^{-1} are caused by the CO_2 in the air. In addition, the appearance of the C=O and (-COOH-) stretching mode indicates that there exists unabsorbed oleic acid in the as-prepare 5 nm ceria sample. When the sample was annealed at 200 ^{o}C, the oleic acid was incompletely oxidized with some wide peaks on the FT-IR spectrum. After annealing at 350 ^{o}C, the capping agent was completely removed ultimately.

Supplementary Note 2

The XPS study was carried out on all the samples at room temperature to identify cerium valence and oxygen vacancies. The core-level spectra of Ce 3*d* are generally characterized by complex features because of final state effects on the hybridization of the Ce 4*f* orbitals with O 2*p* orbitals and fractional occupancy of the valence 4*f* orbitals[2]. The Ce^{3+} can be easily distinguished by the four yellow peaks corresponding to split $3d_{5/2}$ and $3d_{3/2}$ core holes, and the relative weight between Ce^{3+} and Ce^{4+} components can be provided by a semi-quantitative analysis of the integrated peak area[3].

Supplementary Tables

Supplementary Table 1 | The unit cell parameters (*a*-axis) at different temperatures for 5 nm, 9 nm, 18 nm and bulk ceria obtained using XRD Rietveld refinement. In order to satisfy the demands of the analysis and also limited by the status of the instrument, we have different temperature points for different samples.

T (°C)	5 nm (Å)	9 nm(Å)	18 nm (Å)	bulk (Å)
**-150**	5.40894 (6)	----	5.40595 (8)	5.40377 (4)
**-135**	----	5.40668 (4)	----	----
**-100**	5.41124 (6)	5.40872 (4)	5.40751 (8)	5.40526 (4)
**-50**	5.41280 (7)	5.41068 (4)	5.40991 (8)	5.40711 (4)
**-25**	5.41354 (7)	----	----	----
**0**	5.41297 (7)	5.41141 (4)	5.41072 (6)	5.40914 (4)
**25**	5.41231 (6)	5.41138 (4)	5.41118 (6)	4.41049 (4)
**50**	5.41182 (7)	5.41124 (5)	5.41198 (6)	5.41184 (4)
**75**	5.41108 (7)	5.41154 (5)	5.41240 (6)	5.41311 (4)
**100**	5.41166 (7)	5.41204 (5)	5.41332 (6)	5.41468 (3)
**150**	5.41388 (7)	5.41329 (5)	5.41527 (6)	5.41758 (3)
**200**	5.41519 (7)	5.41495 (5)	5.41716 (6)	5.42042 (3)
**250**	5.41708 (7)	5.41707 (5)	5.41932 (6)	5.42351 (3)
**300**	5.41930 (7)	5.41917 (5)	5.42219 (6)	5.42657 (3)
**350**	5.42120 (7)	5.42193 (5)	5.42530 (6)	5.42959 (3)

Supplementary Table 2 | Lattice constants as well as the oxygen coordinates (O_z) of 5 nm ceria extracted from the low-*r* nPDF refinement. All the PDF results over the entire temperature range are given within tetragonal model in order to obtain continuous structural evolution. When $c_t \approx \sqrt{2}\, a_t$ and $O_z \approx 0.75$, the nanosized ceria could be regarded as the cubic phase.

T (°C)	a_t (Å)	$\sqrt{2}\, a_t$	c (Å)	O_z	R_w (%)
**-150**	3.8175 (30)	5.3987 (42)	5.4260 (52)	0.7578 (9)	9.9
**-100**	3.8184 (32)	5.4001 (45)	5.4323 (58)	0.7571 (9)	10.1
**-50**	3.8204 (31)	5.4029 (44)	5.4323 (54)	0.7572 (10)	10.3
**-25**	3.8210 (33)	5.4038 (47)	5.4348 (60)	0.7573 (9)	10.4
**0**	3.8215 (38)	5.4045 (54)	5.4299 (72)	0.7554 (15)	11.2
**25**	3.8228 (34)	5.4062 (48)	5.4218 (62)	0.7543 (13)	10.8
**50**	3.8246 (29)	5.4088 (41)	5.4147 (50)	0.7518 (12)	10.6
**75**	3.8259 (35)	5.4106 (49)	5.4082 (66)	0.7501 (13)	10.8
**125**	3.8271 (36)	5.4123 (51)	5.4107 (68)	0.7497 (13)	10.8
**225**	3.8302 (38)	5.4167 (54)	5.4159 (72)	0.7493 (11)	10.7

Supplementary References

1. Shukla, N., Liu, C., Jones, P. M. & Weller, D. FTIR study of surfactant bonding to FePt nanoparticles. *J. Magn. Magn. Mater.* **266**, 178-184 (2003).
2. Mullins, D., Overbury, S. & Huntley, D. Electron spectroscopy of single crystal and polycrystalline cerium oxide surfaces. *Surf. Sci.* **409**, 307-319 (1998).
3. Deshpande, S., Patil, S., Kuchibhatla, S. V. & Seal, S. Size dependency variation in lattice parameter and valency states in nanocrystalline cerium oxide. *Appl. Phys. Lett.* **87**, 133113 (2005).
